# The feeding behaviour of Amyotrophic Lateral Sclerosis mouse models is modulated by the Ca^2+^‐activated K_Ca_3.1 channels

**DOI:** 10.1111/bph.15665

**Published:** 2021-10-05

**Authors:** Germana Cocozza, Stefano Garofalo, Marta Morotti, Giuseppina Chece, Alfonso Grimaldi, Mario Lecce, Ferdinando Scavizzi, Rossella Menghini, Viviana Casagrande, Massimo Federici, Marcello Raspa, Heike Wulff, Cristina Limatola

**Affiliations:** ^1^ Instituto di Ricovero e Cura a Carattere Scientifico (IRCCS) Neuromed Pozzilli Italy; ^2^ Department of Physiology and Pharmacology Sapienza University of Rome Rome Italy; ^3^ Center for Life Nanoscience Istituto Italiano di Tecnologia@Sapienza Rome Italy; ^4^ Department of Molecular Medicine Sapienza University of Rome Rome Italy; ^5^ EMMA CNR Monterotondo Italy; ^6^ Department of Systems Medicine Tor Vergata University of Rome Rome Italy; ^7^ Department of Pharmacology University of California, Davis Davis California USA

**Keywords:** CSF, hypothalamus feeding behaviour, ion channels, microglia, neurodegenerative disease, neuroinflammation

## Abstract

**Background and Purpose:**

Amyotrophic lateral sclerosis (ALS) patients exhibit dysfunctional energy metabolism and weight loss, which is negatively correlated with survival, together with neuroinflammation. However, the possible contribution of neuroinflammation to deregulations of feeding behaviour in ALS has not been studied in detail. We here investigated if microglial K_Ca_3.1 is linked to hypothalamic neuroinflammation and affects feeding behaviours in ALS mouse models.

**Experimental Approach:**

hSOD1^G93A^ and TDP43^A315T^ mice were treated daily with 120 mg·kg^−1^ of TRAM‐34 or vehicle by intraperitoneal injection from the presymptomatic until the disease onset phase. Body weight and food intake were measured weekly. The later by weighing food provided minus that left in the cage. RT‐PCR and immunofluorescence analysis were used to characterize microglia phenotype and the main populations of melanocortin neurons in the hypothalamus of hSOD1^G93A^ and age‐matched non‐tg mice. The cannabinoid–opioid interactions in feeding behaviour of hSOD1^G93A^ mice were studied using an inverse agonist and an antagonist of the cannabinoid receptor CB_1_ (rimonabant) and μ‐opioid receptors (naloxone), respectively.

**Key Results:**

We found that treatment of hSOD1^G93A^ mice with the K_Ca_3.1 inhibitor TRAM‐34 (i), attenuates the pro‐inflammatory phenotype of hypothalamic microglia, (ii) increases food intake and promotes weight gain, (iii) increases the number of healthy pro‐opiomelanocortin (POMC) neurons and (iv), changes the expression of cannabinoid receptors involved in energy homeostasis.

**Conclusion and Implications:**

Using ALS mouse models, we describe defects in the hypothalamic melanocortin system that affect appetite control. These results reveal a new regulatory role for K_Ca_3.1 to counteract weight loss in ALS.

**Abbreviations**
ALS: amyotrophic lateral sclerosis
BMI: body mass index
IBA1: ionized calcium‐binding adaptor molecule 1
POMC: pro‐opiomelanocortin
α‐MSH: α‐melanocyte‐stimulating hormone
tg: transgenic

**What is already known**

- We recently demonstrated the involvement of K_Ca_3.1 in microglia activation in ALS.
- The K_Ca_3.1 blocker, senicapoc, is safe and well tolerated in clinical trials.

**What does this study add**

- New insights regarding the importance of counteracting inflammation to alleviate weight loss in ALS.
- Cannabinoid–opioid interactions are crucial for the hypothalamic regulation of feeding by K_Ca_3.1 channels.

**What is the clinical significance**

- Identification of K_Ca_3.1 channels as key modulators in feeding behaviour of ALS.

## 1 INTRODUCTION

Amyotrophic lateral sclerosis (ALS) is an adult‐onset neurodegenerative disease characterized by degeneration of upper and lower motor neurons, resulting in paralysis, inability to speak and death within 3–5 years after the first symptoms (Talbott et al., 2016). The first ALS‐related gene, encoding a cytosolic Cu/Zn‐binding SOD (*SOD1*) was reported in 1993 (Dal Canto & Gurney, 1994; Ripps et al., 1995; Rosen et al., 1993) and since 2011, mutations in more than 20 genes have been identified (e.g. *C9orf72*, *TARDBP*, *FUS*, *HNRNPA1*, *SQSTM1*, *VCP*, *OPTN* and *PFN1*). The underlying causes of ALS still need to be fully established. With regard to the nutritional aspect, hypermetabolism and low premorbid body mass index (BMI) at diagnosis have been identified as risk factors for ALS (Desport et al., 1999; Marin et al., 2010; Paganoni et al., 2011). ALS patients frequently suffer from weight loss, which is associated with shorter survival (Moglia et al., 2019; Shimizu et al., 2012). Consistently, mild obesity is associated with longer survival of ALS patients (Paganoni et al., 2011) and hypercaloric diets increase survival in both patients and ALS mouse models (Dorst et al., 2020; Dupuis et al., 2004; Ludolph et al., 2020; Wills et al., 2014). Several lines of evidence suggest hypothalamus dysfunctions in ALS (Cykowski et al., 2014; Gorges et al., 2017). However, pioglitazone treatment, which acts on the melanocortin pathway in the arcuate nucleus, failed to stimulate body weight gain in ALS patients and in SOD1 mice, suggesting that a breakdown of central processes that regulate energy homeostasis may contribute to alter appetite and body weight in ALS (Vercruysse et al., 2016). Furthermore, deregulation of the feeding behaviour and activation of microglial cells have been linked to hypothalamic neuroinflammation, leading to opposite results, such as involuntary weight loss or obesity (Ávalos et al., 2018; García‐Cáceres et al., 2018; Thuc et al., 2017). Animals peripherally injected with LPS display a rapid increase in the expression of inflammatory cytokines in the hypothalamus that has been implicated in the generation of the anorexic response (Layé et al., 2000; Wisse et al., 2007). Consumption of a hypercaloric diet affects the number and size of microglia in the arcuate nucleus and the median eminence, before any changes in body weight gain is observed, suggesting a potential role of these cells in metabolic disorders (Valdearcos et al., 2017). These data support the idea that inflammatory pathways may play a causative role in metabolic dysfunction. In familial ALS‐associated *SOD1* mutations and in the corresponding mouse models, microglia acquire an inflammatory phenotype affecting motor neuron death (McGeer & McGeer, 2002), further myeloid cells expressing mutated *SOD1* promote neurotoxicity (Boillée, 2006). In another ALS mouse model based on *TDP‐43* mutations, the TDP43^A315T^ mice showed pathological aggregates of ubiquitinated proteins in specific neurons and reactive gliosis, with the loss of both upper and lower motor neurons (Wegorzewska, 2009). Recently, we have demonstrated the involvement of the Ca^2+^‐activated potassium channel K_Ca_3.1 in microglia activation in ALS (Cocozza et al., 2018). In the CNS, K_Ca_3.1 channels are expressed by microglial cells, where they regulate cell migration, inflammatory cytokine production and phagocytic activity in physiological and pathological conditions such as glioma, ischaemia, spinal cord injury and Alzheimer's disease (Chen et al., 2011; D'Alessandro et al., 2013; Grimaldi et al., 2016; Jin et al., 2019). Previously, we found that K_Ca_3.1 inhibition in hSOD1^G93A^ mice attenuates the pro‐inflammatory phenotype of microglia in the spinal cord, reduces motor neuron death, delays the onset of muscle weakness and increases survival (Cocozza et al., 2018). Here, we focus our attention on the most important brain region involved in the central control of feeding and energy expenditure, the hypothalamus. We treated hSOD1^G93A^ mice with the selective K_Ca_3.1 inhibitor TRAM‐34 starting at the presymptomatic stage, to investigate a possible link between microglial inflammatory signalling and alteration of the feeding behaviour. We found that chronic inhibition of K_Ca_3.1 activity attenuates the pro‐inflammatory phenotype of microglia in the hypothalamus, inducing the recovery of melanocortin tone and an increase of food consumption and weight gain. Cannabinoid CB_1_ receptors and μ‐opioid receptors regulate energy balance via multiple neural pathways, promoting food intake and reward (Bermudez‐Silva et al., 2012; Koch et al., 2015). Here, we suggest that these positive effects on feeding behaviour are mediated by CB_1_ receptor activation, which increases ß‐endorphin release by pro‐opiomelanocortin (POMC) neurons.

## 2 METHODS

### 2.1 Animal model

All experiments and procedures were approved by the Italian Ministry of Health (Authorization No. 78/2017‐PR issued 25 January 2017) in accordance with the ethical guidelines on use of animals from the EC Council Directive 2010/63/EU and from the D. Lgs 26/2014. Animal studies are reported in compliance with the ARRIVE guidelines (Percie du Sert et al., 2020) and with the recommendations made by the *British Journal of Pharmacology* (Lilley et al., 2020). All possible efforts were made to minimize animal suffering and to reduce the number of animals used per condition by calculating the necessary sample size before performing the experiments. hSOD1^G93A^ transgenic mice, which express 20 copies of mutant human SOD1^G93A^ [B6.Cg‐Tg (SOD1‐G93A)1Gur/J line] and hemizygous TDP43^A315T^ male mice [B6.Cg‐Tg (Prnp‐TARDBP*A315T)95Balo/J] (Cat# JAX:010700, RRID:IMSR_JAX:010700), were obtained from The Jackson Laboratory (Bar Harbor, ME, USA) (RRID:IMSR_JAX:004435) (Charles River, Calco, Italy). B6.Cg‐Tg (SOD1‐G93A)1Gur/J were maintained as hemizygotes by breeding transgenic males with wild‐type C57BL/6J females from Charles River Laboratories, both maintained on the C57BL/6J genetic background. Age‐matched sibling non‐transgenic (non‐tg) C57BL/6J mice were always used as controls. Because it has been demonstrated that there is a gender difference in motor impairment and survival in hSOD1^G93A^ mice (Choi et al., 2008), we decided to use only males, in this proof‐of‐concept study. Transgenic mice were identified by PCR on DNA obtained from tail biopsies. Briefly, tail tips were digested (overnight, 58°C) in a buffer containing 100‐mM Tris–HCl pH 8, 0.1% SDS 20, 5‐mM EDTA pH 8, 200‐mM NaCl and 20‐mg·ml^−1^ proteinase K (Ambion–Thermo Fisher) and the extracted genomic DNA was amplified with SsoFast EvaGreen Supermix (Bio‐Rad, CA) using the following primers:‐ SOD1 forward 5′‐CATCAGCCCTAATCCATCTGA‐3′; SOD1 reverse 5′‐CGCGACTAACAATCAAAGTGA‐3′. Animals were housed at constant temperature (22 ± 1°C) and relative humidity (50%) and were kept on a 12‐h light cycle (light 7:00 AM to 7:00 PM). Food and tap water were freely available. Before the experiments, the mice were randomly assigned into groups. Each experiment involved different animal groups. Transgenic animals were weighted two times a week, beginning at 7 weeks of age. Starting at 6 weeks of age, the mice were evaluated for signs of motor deficit with a behavioural score system. Neurological score was based on the scale originally described by Dal Canto and Gurney (1994) and modified by Weydt et al. (2003).

### 2.2 Mice treatment

Male transgenic animals were weighted once a week, beginning at 6 weeks of age until 16 weeks of age. Starting at 7 weeks of age hSOD1^G93A^, TDP43^A315T^ or non‐tg C57BL/6J mice were randomly grouped into vehicle and TRAM‐34 (1‐[(2‐chlorophenyl)diphenylmethyl]‐1*H*‐pyrazole. Mice were daily treated with 120 mg·kg^−1^ of TRAM‐34 or the same amount of vehicle (50 μl, peanut oil, Sigma‐Aldrich) by intraperitoneal injections. We used 120 mg·kg^−1^ of TRAM‐34 because the circulating half‐life of this compound in mice is 1 h (D'Alessandro et al., 2013). TRAM‐34 was synthesized as previously described (Wulff et al., 2000). The long‐term treatment is not toxic and does not induce changes in body weight, haematology, blood chemistry or necropsy of any major organs, in either mice or rats (Toyama et al., 2008; Wulff et al., 2000). The treatment regimen was chosen to reach a CNS concentration of TRAM‐34 that effectively inhibits K_Ca_3.1 channels, as previously described (Chen et al., 2011). The CB_1_ receptor antagonist/inverse agonist rimonabant (SR141716, Cayman Chemical Company) was dissolved in 5% Tween 80 and 5% polyethylene glycol/saline. Mice were daily treated with 3 mg·kg^−1^ of rimonabant or the same amount of vehicle (saline) by intraperitoneal injections. Animals were treated until the age described in the text. The μ‐opioid receptor antagonist, naloxone hydrochloride (Tocris Bioscience) was dissolved in saline and stored at room temperature until day of use. Mice were daily treated with 7.5 mg·kg^−1^ of naloxone or the same amount of vehicle (saline) by intraperitoneal injections. Animals were treated until the age described in the text.

### 2.3 Food intake and body weight

Mice were singly housed in regular polycarbonate cages (30 × 16 × 11 cm), at constant temperature (22 ± 1°C) and humidity (50%), and were kept on a 12‐h light cycle (light 7:00 AM to 7:00 PM). Nesting objects were included with bedding (sawdust) materials. Food (regular chow, containing 14% protein, 5% fat and 3041 kcal/kg) intake was measured weekly, weighing food provided and left in the cage. The same food was provided to all the mouse strains used in these experiments. For this experiment, intraperitoneal injections were performed at the end of the light phase, between 6:00 AM and 7:00 PM, and food was weighted after 16 h. Transgenic animals were weighed once a week from 6 until 16 weeks of age.

### 2.4 Energy balance

The experiments of indirect calorimetry were performed using the LabMaster system (TSE Systems, Bad Homburg, Germany). Mice were acclimatized into the metabolic cages for 24 h before starting the experiments. The measurements were taken every 15 min along a total 12‐h period. Oxygen consumption (*V*O_2_) is expressed as millilitres of O_2_ consumed per kilogram of body weight per minute. Carbon dioxide production (*V*CO_2_) is expressed as millilitres of CO_2_ produced per kilogram of body weight per minute. The respiratory quotient is defined as *V*CO_2_/*V*O_2_ ratio.

### 2.5 Hypothalamic neuronal cultures

Neuronal cultures were obtained from the hypothalamus of Postnatal Day 0–1 (p0–p1) C57BL/6 mice. Mice were killed by cervical dislocation and the hypothalami were removed and tissues were digested with 30‐U·ml^−1^ papain (Sigma‐Aldrich) at 37°C. After 20 min, the reaction was stopped by removing papain and washing twice with 2 ml of prewarmed Basal Medium Eagle (BME). Tissue was then triturated with a glass pipette to obtain single‐cell suspensions, which were applied to 100 μm per 40‐μm cell strainers. Cells were plated at a density of 250,000 cells per well in 24‐well plates (1.9 cm^2^ per well culture area) with BME. The culture medium was changed completely and carefully after 4 h with neurobasal medium (Gibco, Thermo Fisher) (2‐mM glutamine, 1% B27, 100‐U·ml^−1^ penicillin and 0.1‐mg·ml^−1^ streptomycin). Half of the culture media was removed and replaced with fresh media every 3 days. After 9 days in culture, purity of neuronal cells, analysed as anti‐tubulin β3 (TUBB3) (Covance), ranges between 60% and 70%.

### 2.6 Primary microglial cultures

Microglia cultures were obtained from mixed glia cultures derived from the cerebral cortices of p0–p1 C57BL/6 mice, as described (Lauro et al., 2010). In brief, cortices were chopped and digested in 15‐U·ml^−1^ papain for 20 min at 37°C. Cells (5 × 10^5^ cells·cm^−2^) were plated on flasks coated with poly‐l‐lysine (100 mg·ml^−1^) in DMEM supplemented with 10% FBS, 100‐U·ml^−1^ penicillin and 0.1‐mg·ml^−1^ streptomycin. After 7–9 days, cells were shaken for 2 h at 37°C to detach and collect microglial cells. These procedures gave almost pure microglial cell populations, as verified by immunofluorescence staining using GFAP‐ and ionized calcium‐binding adaptor molecule 1 (IBA1)‐specific antibodies (Lauro et al., 2010).

### 2.7 Microglia/hypothalamic neuron co‐cultures

After 7–9 days in culture, hypothalamic neurons (250,000 cells per well) were co‐cultured with primary microglia (pretreated with 2.5‐μM TRAM‐34, or DMSO, 0.01%, Sigma‐Aldrich, USA, as vehicle) plated (100,000 cells per well) on poly‐l‐lysine‐coated transwells (Corning, Sigma‐Aldrich, USA, 0.4‐μm pore size). After 24 h of co‐culture, hypothalamic neurons were lysed in TRIzol reagent for isolation of RNA.

Isolation of CD11b^+^ cells from hypothalamus: Non‐tg, vehicle and TRAM‐34‐treated hSOD1^G93A^ mice were deeply anaesthetized with chloral hydrate (400 mg· kg^−1^, i.p.) and decapitated. Brains were removed, hypothalamic tissues were cut into small pieces and single‐cell suspension was achieved in HBSS. The tissue was further mechanically dissociated using a glass wide‐tipped pipette and the suspension was applied to a 30‐μm cell strainer (Miltenyi Biotec). Cells were processed immediately for MACS MicroBead separation. CD11b^+^ cells were magnetically labelled with CD11b MicroBeads. The cell suspension was loaded onto a MACS Column placed in the magnetic field of a MACS Separator and the negative fraction was collected. After removing the magnetic field, CD11b^+^ cells were eluted as a positive fraction.

### 2.8 Real‐time PCR

Hypothalamic tissues and CD11b^+^ cells of non‐tg, vehicle and TRAM‐34‐treated hSOD1^G93A^ mice were lysed in TRIzol reagent (Invitrogen) for isolation of RNA. The quality and yield of RNAs were verified using the NANODROP One system (Thermo Scientific). Reverse transcription reaction was performed in a thermocycler (MJ Mini Personal Thermal Cycler; Bio‐Rad), using iScript Reverse Transcription Supermix (Bio‐Rad) according to the manufacturer's protocol, under the following conditions: incubation at 25°C for 5 min, reverse transcription at 42°C for 30 min and inactivation at 85°C for 5 min. RT‐PCR of genes described was carried out in a I‐Cycler IQ Multicolor RT‐PCR Detection System using SsoFast EvaGreen Supermix (Bio‐Rad). The PCR protocol consisted of 40 cycles of denaturation at 95°C for 30 s and annealing/extension at 60°C for 30 s. Relative gene expression was calculated by ΔΔCT analysis relative to *Gapdh* expression levels: *Gapdh*: forward (F), 5′‐TCGTCCCGTAGACAAAATGG‐3′, reverse (R), 5′‐TTGAGGTCAATGAAGGGGTC‐3′; *Il‐1ß* : (F), 5′‐GCAACTGTTCCTGAACTCAACT‐3′, (R), 5′‐ATCTTTTGGGGTCCGTCAACT‐3′; *Nos2* : (F), 5′‐ACATCGACCCGTCCACAGTAT‐3′, (R) 5′‐CAGAGGGGTAGGCTTGTCTC‐3′; *Tnf* : (F), 5′‐GTGGAACTGGCAGAAGAG‐3′, (R) 5′‐CCATAGAACTGATGAGAGG‐3′; *Bdnf* : (F), 5′‐CGGC GCCCATGAAAGAAGTA‐3′, (R), 5′‐AGACCTCTCGAA CCTGCCCT‐3′; *Chil3*: (F) 5′‐CAGGTCTGGCAATTCTTCTGAA‐3′, (R) 5′‐GTCTTGCTCATGTGTGTAAGTGA‐3′; Kccn4: (F) 5′‐GGCTGAAACACCGGAAGCTC‐3′, (R) 5′‐CAGCTCTGTCAGGGCATCCA‐3′; *P2ry12* : (F) 5′‐CCTGTCGTCAGAGACTACAAG‐3′, (R) 5′‐GGATTTACTGCGGATCTGAA‐3′; *Pomc*: (F), 5′‐AGTGCCAGGACCTCACCA‐3′, (R) 5′‐CAGCGAGAGGTCGAGTTTG‐3′; *Mc4r* : (F), 5′‐ATGGCATGTATACTTCCCTCCA‐3′, (R), 5′‐CCTCCCAGAGGATAGAAACAGA‐3′; *Agrp* : (F), 5′‐CAGGCTCTGTTCCCAGAGTT‐3′, (R), 5′‐TCTAGCACCTCCGCCAAA‐3′; *Cnr1*: (F), 5′‐GGCACCTCTTTCTCAGTCACGT‐3′, (R), 5′‐GGTGATGGTACGGAAGGTGGTA‐3′; *Oprm1*: (F), 5′‐CCCTCTATTCTATCGTGTGTGT‐3′, (R), 5′‐AGAAGAGAGGATCCAGTTGCA‐3′; *Apoe*: (F), 5′‐TGTGGGCCGTGCTGTTGGTC‐3′, (R), 5′‐GCCTGCTCCCAGGGTTGGTTG‐3′; *Cst7*: (F), 5′‐CCTGCCTTGAAGCGGACTC‐3′, (R), 5′‐CACCTCAAAACTGTGGAGCCA‐3′; *Lpl*: (F), 5′‐AGACTCGCTCTCAGATGCCCT‐3′, (R), 5′‐GCTTGCCATCCTCAGTCCC‐3′; and *Trem2*: (F), 5′‐ATGGGACCTCTCCACCAGTT‐3′, (R), 5′‐TCACGTACCTCCGGGTCCA‐3′.

### 2.9 Immunofluorescence

Hypothalamic slices were prepared from hSOD1^G93A^ or non‐tg mice treated with vehicle or TRAM‐34 as described. Hypothalamic sections (20 μm) were washed in PBS; blocked (3% goat serum in 0.3% Triton X‐100) for 1 h, at RT; and incubated overnight at 4°C with specific antibodies diluted in PBS containing 1% goat serum and 0.1% Triton X‐100. The sections were incubated with the following primary Abs: IBA1 (Wako, 1:500), POMC (Abcam, 1:200) and GFAP (Novus Biologicals, 1:500). After several washes, sections were stained with fluorophore‐conjugated secondary antibody and Hoechst for nuclei visualization and analysed using a fluorescence microscope. For IBA1 staining, coronal sections were first boiled for 20 min in citrate buffer (pH 6.0) at 95–100°C. For Pomc staining, coronal sections were first boiled for 20 min in Tris‐EDTA buffer (pH 9.0) at 95–100°C. At least three animals per condition were analysed (12 serial coronal sections for each animal, in each group, covering the entire arcuate nucleus segment). Mice were randomly selected from the treatment groups. Images were digitized using a CoolSNAP camera (Photometrics, Tucson, USA) coupled to an ECLIPSE Ti‐S microscope (Nikon, Tokyo, Japan) and processed using MetaMorph7.6.5.0 image analysis software (Molecular Devices, San Jose, USA). Signal co‐localization was analysed measuring the average fluorescence intensity (pixels) of merged signals. The Immuno‐related procedures used comply with the recommendations made by the *British Journal of Pharmacology* (Alexander et al., 2019).

### 2.10 Skeleton analysis

Microglia morphology was obtained by confocal microscopy using IBA1 signal. Twenty‐micrometre z‐stacks were acquired at 0.5‐μm intervals using an FV10i laser scanning microscope (Olympus, Tokyo, Japan) at 60× objective. Cell morphology was measured using a method adapted from that described by Morrison and Filosa (2013). Maximum intensity projections for the IBA1 channel of each image were generated, binarized and skeletonized using the Skeletonize 2D/3D plugin in ImageJ, after which the Analyze Skeleton plugin (http://imagej.net/AnalyzeSkeleton) was applied. The average branch number (process end points per cell) and length per cell were recorded for each image with a voxel size exclusion limit of 150 applied. The areas of the soma and scanning domain were measured for each cell.

### 2.11 CSF collection and neuropeptide analysis

For collection of CSF (Lim et al., 2018), mice were deeply anaesthetized with chloral hydrate (400 mg·kg^−1^, i.p.) before being placed on the stereotaxic apparatus under a dissecting microscope, monitoring the temperature and the spontaneous breathing. Once the dura over the cisterna magna was exposed (triangular in shape with usually one to two large blood vessels running through the area; either side of or between the blood vessels is optimal for capillary insertion and CSF collection), we used a microscope to see the sharpened point of the capillary puncture into the cisterna magna. CSF could be automatically drawn into the capillary tube once the opening had been punctured, and the mice were killed after sampling of CSF by anaesthetic overdose. CSF samples were collected in 1.5‐ml microtubes with 1 μl of 20× protease inhibitor (Roche) and then quickly centrifuged to mix the sample with the protease inhibitor at the bottom of the collection tube. The MILLIPLEX MAP Rat/Mouse Neuropeptide Magnetic Bead Panel (Merck) was used for the simultaneous quantification of α‐melanocyte‐stimulating hormone (α‐MSH) and β‐endorphin in the CSF. Samples were mixed with antibody‐linked magnetic beads on 96‐well filter‐bottom plates and incubated at room temperature for 2 h followed by overnight incubation at 4°C. Room temperature incubation steps were performed on an orbital plate shaker at 100× *g*. Plates were washed twice with wash buffer on magnetic plate washer and then incubated with biotinylated detection antibody for 2 h at room temperature. Samples were washed twice as above and suspended in streptavidin–PE. After incubation for 40 min at room temperature, two additional washes were performed, and the samples were resuspended in Reading Buffer. Plates were read using a Luminex‐200 instrument. The median fluorescence intensity (MFI) data were analysed using a weighted five‐parameter logistic or spline curve‐fitting method for calculating analyte concentrations in samples.

### 2.12 Measurement of β‐endorphin by elisa

The hypothalamus of hSOD1^G93A^ mice treated with vehicle or TRAM‐34 + rimonabant was disrupted with a homogenizer and analysed for β‐endorphin content using a commercially available β‐endorphin elisa kit, in accordance to manufacturer's instructions (MyBioSource). Briefly, after two freeze–thaw cycles were performed to break the cell membranes, the homogenates were centrifuged for 5 min at 5000× *g*, 2–8°C. The supernatant was removed and assayed immediately. BCA Reagent Kit was used to measure protein concentration in samples (Thermo Scientific) and β‐endorphin content was calculated as pg·ml^−1^.

### 2.13 Data and statistical analysis

Data are shown as the mean ± SEM. Statistical significance was assessed by unpaired Student's *t*‐test, one‐way ANOVA or two‐way ANOVA for parametrical data, as indicated; Holm–Sidak test was used as a post hoc test; Mann–Whitney rank test and Kruskal–Wallis for non‐parametrical data, followed by Dunn's or Tukey's post hoc tests. A *P* value <0.05 was considered to be statistically significant. Statistical analysis was done only for the experimental groups where *n* = 5 or greater per group. All experiments are performed using randomization and blinding analysis. The exact group size for each experimental group was provided in the figure legends. For each experiment, the sample size (*n*) was chosen considering the following relation: nZ2sigma (Zalpha/D)2, where sigma is substituted by an estimate of variance (s2); alpha is at 0.05 (and Zalpha = −2), and D is the difference among treatments. Criteria of animal exclusion/inclusion were pre‐established; animals considered for the analysis were selected for age. At weaning, pups from different colonies were mixed together and mice were randomly treated with TRAM‐34 or vehicle. Group size is the number of independent animals or cell cultures, and statistical analysis was performed using these independent values. All statistical analyses were carried out using the SigmaPlot 11.0 software (Systat Software GmbH, Erkrath, Germany). The data and statistical analysis comply with the recommendations of the *British Journal of Pharmacology* on experimental design and analysis in pharmacology (Curtis et al., 2018).

### 2.14 Materials

Culture media, fetal bovine serum (FBS), goat serum, penicillin G, streptomycin, glutamine, Na pyruvate, TRIzol reagent (#T9424), ThermoScript RT‐PCR System and Hoechst (#33342, RRID:AB_10626776) were from Gibco Invitrogen (Carlsbad, CA, USA). Poly‐l‐lysine‐coated transwells (#3412), peanut oil (#P2144) and papain (#P3125) were from Sigma‐Aldrich, USA. Rimonabant (SR141716, #9000484) was from Cayman Chemical Company. Naloxone was from Tocris Bioscience (#0599) and TRAM‐34 (1‐[(2‐chlorophenyl)diphenylmethyl]‐1*H*‐pyrazole has been kindly provided by Dr Heike Wulff, Department of Pharmacology, University of California, Davis, Davis, CA, USA. Food (#X.O4.20.1.0017) was from A.CO srl, Varallo Pombia, Italy. Anti‐Iba1 (#019‐19741, RRID:AB_839504) was from Wako, Osaka, Japan; anti‐POMC ([2F6H11] ab73092, RRID:AB_1267621) was from Abcam, Cambridge, UK; anti‐glial fibrillary acidic protein (GFAP; #NB300‐141, RRID:AB_10001722) was from Novus Biologicals, Littleton, USA; and anti‐tubulin β3 (TUBB3) (#MMS‐435P, RRID:AB_2313773) was from Covance, Princeton, USA. Secondary Abs were from Dako (Milan, Italy). The MILLIPLEX MAP Rat/Mouse Neuropeptide Magnetic Bead Panel (#RMNPMAG‐83K) was from Merck, Milan, Italy. Beta‐Endorphin elisa kit (#BS703919) was from MyBioSource, San Diego, CA, USA. BCA Reagent Kit (#23225) and proteinase K (#Am‐2548) were from Ambion–Thermo Fisher, Germany. SsoFast EvaGreen Supermix (#172‐5201) was from Bio‐Rad, CA, USA

### 2.15 Nomenclature of targets and ligands

Key protein targets and ligands in this article are hyperlinked to corresponding entries in the IUPHAR/BPS Guide to PHARMACOLOGY http://www.guidetopharmacology.org and are permanently archived in the Concise Guide to PHARMACOLOGY 2019/20 (Alexander et al., 2019).

## 3 RESULTS

### 3.1 K_Ca_3.1 channels modulate hypothalamic microglia in hSOD1^G93A^ mice

**FIGURE 1:**
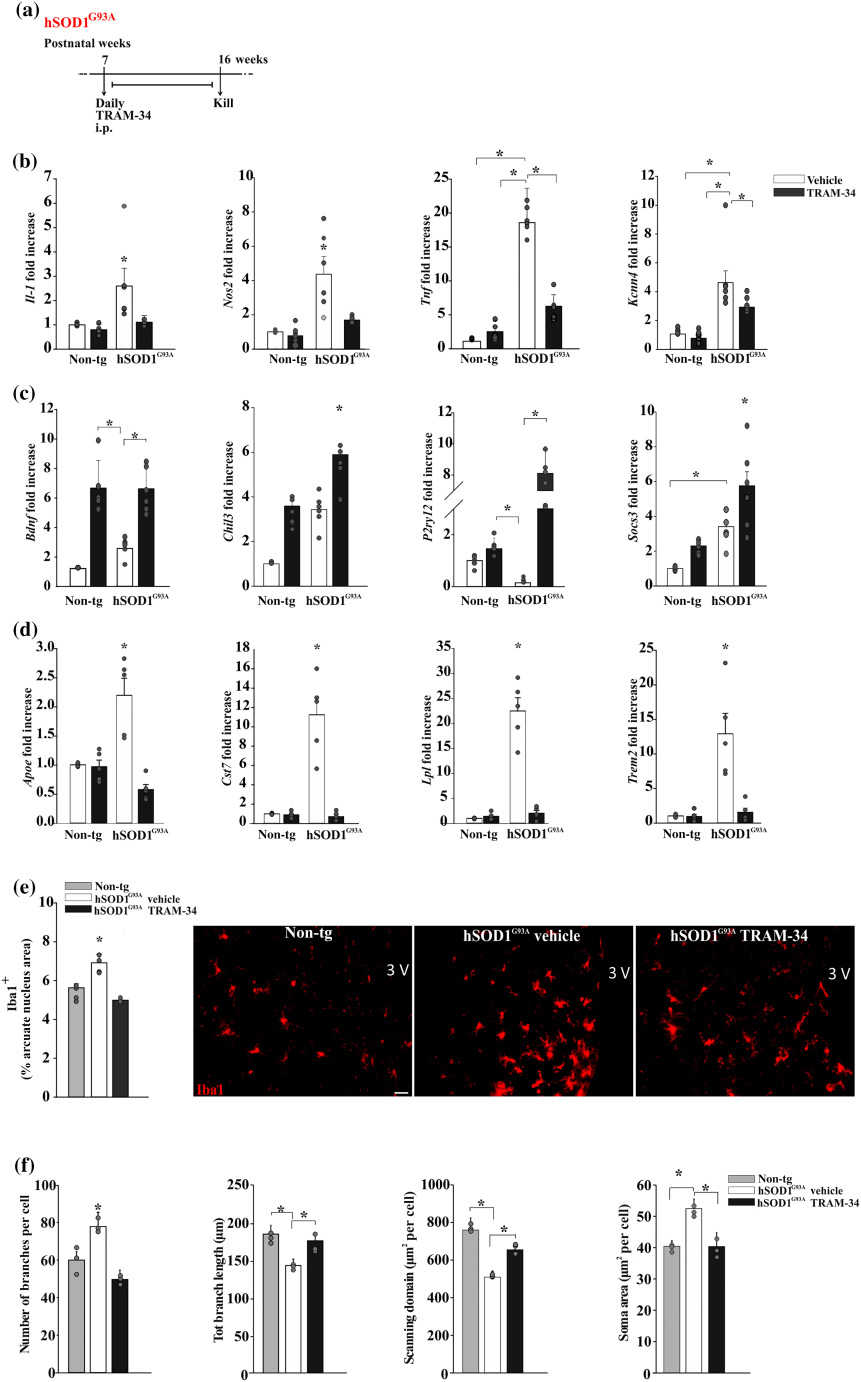
K_Ca_3.1 channels modulate hypothalamic microglia in hSOD1^G93A^. (a) Experimental scheme. (b,c) RT‐PCR analyses of the relative expression level of (b) pro‐inflammatory (top) and (c) anti‐inflammatory (bottom) genes in CD11b^+^ cells isolated from the hypothalamus of non‐tg or hSOD1^G93A^ mice (16 weeks) treated with vehicle or TRAM‐34; (*n* = 6 mice). Data are mean ± SEM. ^*^ *P* < 0.05 one‐way ANOVA. (d) RT‐PCR analyses of the relative expression level of neurodegeneration‐associated genes in CD11b^+^ cells isolated from the hypothalamus of non‐tg or hSOD1^G93A^ mice (16 weeks) treated with vehicle or TRAM‐34; (*n* = 5 mice). Data are mean ± SEM. ^*^ *P* < 0.05, one‐way ANOVA. (e) Mean (±SEM) area of Iba1^+^ cells (expressed as % of hypothalamic arcuate nucleus (ARC) area) in non‐tg or hSOD1^G93A^ mice (16 weeks) treated with vehicle or TRAM‐34. Mice were randomised to three groups (*n* = 5 per group). ^*^ *P* < 0.05, one‐way ANOVA. Right: representative immunofluorescence images. Scale bar: 20 μm. (f) Quantification of branch numbers and length, soma and scanning domain area of Iba1^+^ cells in slices obtained from the hypothalamic ARC of non‐tg and hSOD1^G93A^ mice (16 weeks) treated with vehicle or TRAM‐34. Bottom: representative images (original magnification, ×600; scale bar: 10 μm) (*n* = 5 mice per treatment, 20 cells per mice in at least six slices). ^*^ *P* < 0.05, one‐way ANOVA. Error bars show mean ± SEM

It has been shown that central and peripheral inflammation affects the feeding behaviour in animal models (Braun & Marks, 2010). We have previously demonstrated that the Ca^2+^‐activated potassium channel, K_Ca_3.1, modulates the production of inflammatory molecules produced by spinal microglia in hSOD1^G93A^ mice (Cocozza et al., 2018). To investigate whether the K_Ca_3.1 channels are also involved in the feeding behaviour in hSOD1^G93A^ mice by modulating the hypothalamic microglia phenotype, hSOD1^G93A^ and age‐matched non‐tg mice were treated with TRAM‐34 (daily, 120 mg·kg^−1^) from the age of 7 weeks (before appearance of signs) until 16 weeks (when signs are evident) (Figure 1a). After this period, mice were killed and CD11b^+^ cells were isolated from the hypothalamus. RT‐PCR analysis on these cells revealed that K_Ca_3.1 blockade by TRAM‐34, in hSOD1^G93A^ mice, decreased the expression of pro‐inflammatory genes in CD11b^+^ cells (*Nos2*, *Tnf* , *Il‐1ß* and *Kcnn4* , Figure 1b) and increased anti‐inflammatory genes (*Ym1*, *P2yr12* and *Socs3*, Figure 1b, bottom). Note that CD11b^+^ cells from non‐tg mice, upon TRAM‐34 treatment, express higher levels of *Bdnf* (Figure 1c). In these cells, we also found that K_Ca_3.1 inhibition decreased the expression of neurodegeneration‐associated genes, thus preventing the switch from a homeostatic to a neurodegenerative phenotype (Figure 1d). Next, we investigated if K_Ca_3.1 channel blockade could affect microglial density and morphology in the hypothalamic arcuate nucleus region, using ionized calcium‐binding adaptor molecule 1 (IBA1) as a tool to identify these cells. Microscopy image analysis of the area occupied by IBA1^+^ cells in brain slices of the arcuate nucleus revealed a mean increase in hSOD1^G93A^ mice in comparison with age‐matched non‐tg mice (Figure 1e.). TRAM‐34 treatment reduced the area of IBA1^+^ cells to values similar to non‐tg mice (Figure 1e) (non‐tg 5.63 ± 0.27; hSOD1^G93A^ vehicle 6.98 ± 0.42; and hSOD1^G93A^ TRAM‐34 5.06 ± 0.01; *n* = 5). IBA1^+^ cells in these regions were also analysed for morphology by the skeleton analysis to quantify single‐cell shape. In TRAM‐34‐treated hSOD1^G93A^ mice, IBA1^+^ cells had smaller soma, bigger scanning domain and higher branch length, in comparison with vehicle‐treated mice, suggesting that the treatment reduced microglia activation (Figure 1f).

### 3.2 Inhibition of K_Ca_3.1 leads to weight gain by increasing food consumption in two mouse models of ALS: hSOD1^G93A^ and TDP43^A315T^

**FIGURE 2:**
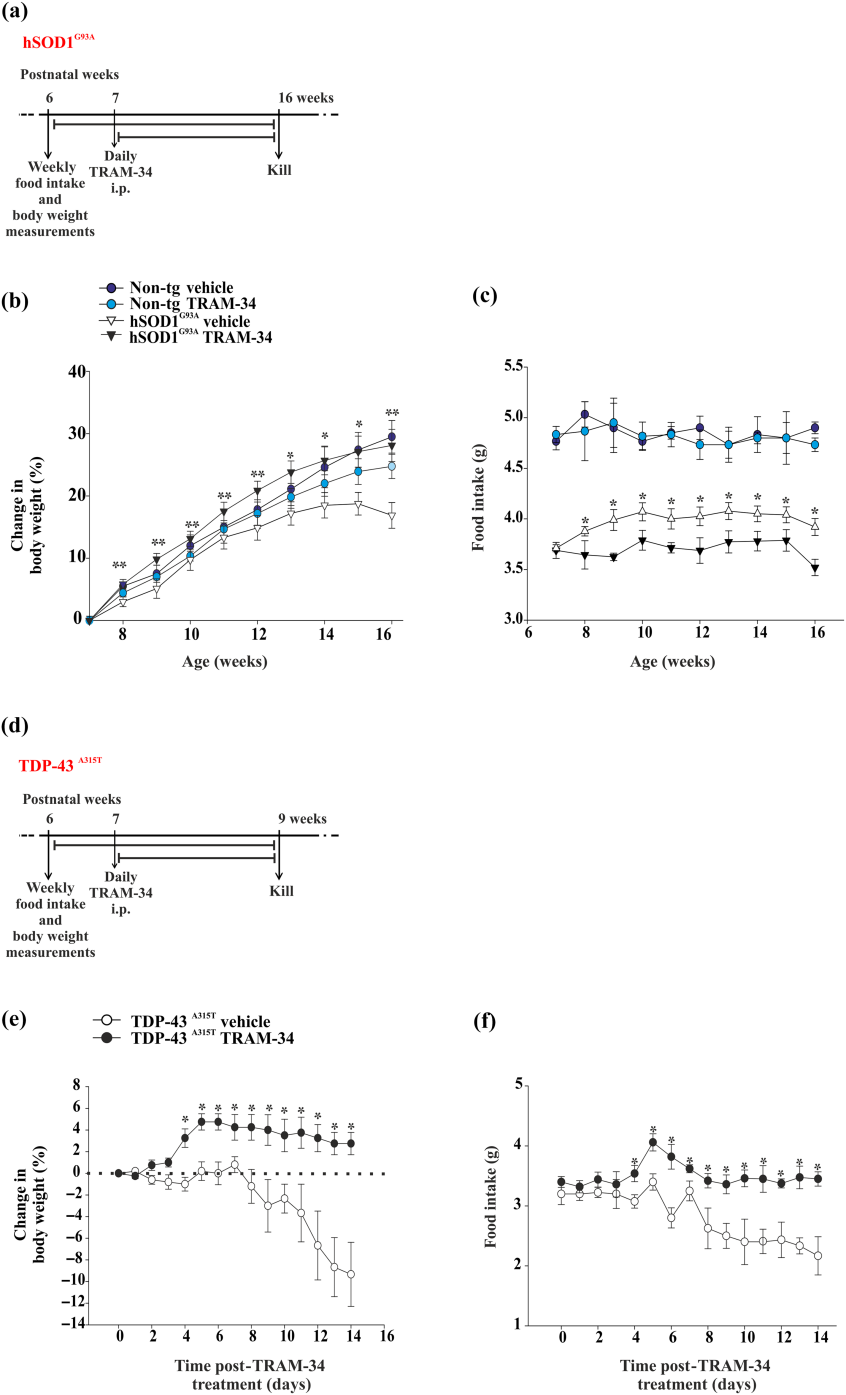
K_Ca_3.1 inhibition increases body weight and food intake in two different ALS mouse models. (a) Experimental scheme. (b) Change in weekly body weight in non‐tg or hSOD1^G93A^ mice (16 weeks) treated with vehicle or TRAM‐34 (non‐tg vehicle/TRAM‐34, *n* = 12; hSOD1^G93A^ vehicle, *n* = 16; and hSOD1^G93A^ TRAM‐34, *n* = 18 mice). Data are the mean ± SEM. ^*^ *P* < 0.05 and ^*^ *P* < 0.05 hSOD1^G93A^ TRAM‐34 versus hSOD1^G93A^ vehicle, one‐way ANOVA; 16 weeks hSOD1^G93A^ TRAM‐34 versus hSOD1^G93A^ vehicle, two‐way ANOVA. (c) Change in weekly food intake in non‐tg or hSOD1^G93A^ mice (16 weeks) treated with vehicle or TRAM‐34 (non‐tg vehicle/TRAM‐34, *n* = 6; hSOD1^G93A^ vehicle/TRAM‐34, *n* = 10). Data are the mean ± SEM. ^*^ *P* < 0.05 hSOD1^G93A^ TRAM‐34 versus hSOD1^G93A^ vehicle, one‐way ANOVA. (d) Experimental scheme. (e) Change in weekly body weight in TDP‐43^A315T^ treated with vehicle or TRAM‐34 (TRAM‐34, *n* = 5; vehicle, *n* = 5). Data are the mean ± SEM. ^*^ *P* < 0.05 versus vehicle Student's *t*‐test. (f) Change in weekly food intake in TDP‐43^A315T^ treated with vehicle or TRAM‐34 (TRAM‐34, *n* = 5; vehicle, *n* = 5). Data are the mean ± SEM. ^*^ *P* < 0.05 versus vehicle Student's *t*‐test

We have previously shown that inhibiting K_Ca_3.1 channels with TRAM‐34 leads to increased body weight in hSOD1^G93A^ mice (Cocozza et al., 2018). We now confirmed these data monitoring body weight variations in age‐matched non‐tg and hSOD1^G93A^ mice upon TRAM‐34 or vehicle treatment from 7 to 16 weeks of age (Figure 2a). As expected, vehicle‐treated hSOD1^G93A^ mice had a slower rate of weight gain in comparison with non‐tg mice, starting from 10 weeks of age (Figure 2b). However, upon TRAM‐34 treatment, hSOD1^G93A^ mice achieved a similar weight as age‐matched non‐tg mice. We hypothesized that the increase in body weight for TRAM‐34‐treated hSOD1^G93A^ mice was due to still unknown effects in stimulating food intake. To test this hypothesis, mice were treated daily (between 5:00 PM and 6:00 PM) with 120 mg·kg^−1^ of TRAM‐34 or the same amount of vehicle (50 μl, peanut oil) and food intake was measured weekly, weighing the food provided and that left in the cage every 16 h throughout all the treatments. Starting at week 8, TRAM‐34‐treated hSOD1^G93A^ mice consumed greater amounts of food in comparison with vehicle‐treated mice (Figure 2c), likely leading to the observed increase in body weight (Figure 2b). No change in food intake was observed upon TRAM‐34 treatment of age‐matched non‐tg mice. These experiments were replicated in a different ALS mouse model based on TDP43 mutations (Wegorzewska, 2009). TDP43^A315T^ mice were treated with TRAM‐34 (daily, 120 mg·kg^−1^) from the age of 7 weeks (before appearance of signs) until 9 weeks (when signs are evident) (Figure 2d). After a few days, TRAM‐34‐treated TDP‐43^A315T^ mice gained more weight (Figure 2e) and consumed more food (Figure 2f) in comparison with vehicle‐treated mice, thus extending the results obtained in hSOD1^G93A^ mice to a different familial ALS model. To evaluate whether K_Ca_3.1 inhibition directly modulates energy expenditure to compensate for weight loss, we measured energy balance using indirect calorimetry in non‐tg and hSOD1^G93A^ mice treated or not with TRAM‐34 for 6 days during the presymptomatic phase. Our preliminary experiments, shown in Figure S2, show that hSOD1^G93A^ mice have a reduced respiratory exchange ratio (RER or *V*CO_2_/*V*O_2_), both during the night and day phases, whereas K_Ca_3.1 inhibition restores the metabolic alterations, suggesting that K_Ca_3.1 channels affect determinant factors of energy metabolism in hSOD^G93A^ mice. However, we cannot exclude that alteration of K_Ca_3.1 channel activity also affected energy homeostasis at cellular level.

### 3.3 K_Ca_3.1 inhibition changes the expression of hypothalamic neuropeptides involved in energy homeostasis

**FIGURE 3:**
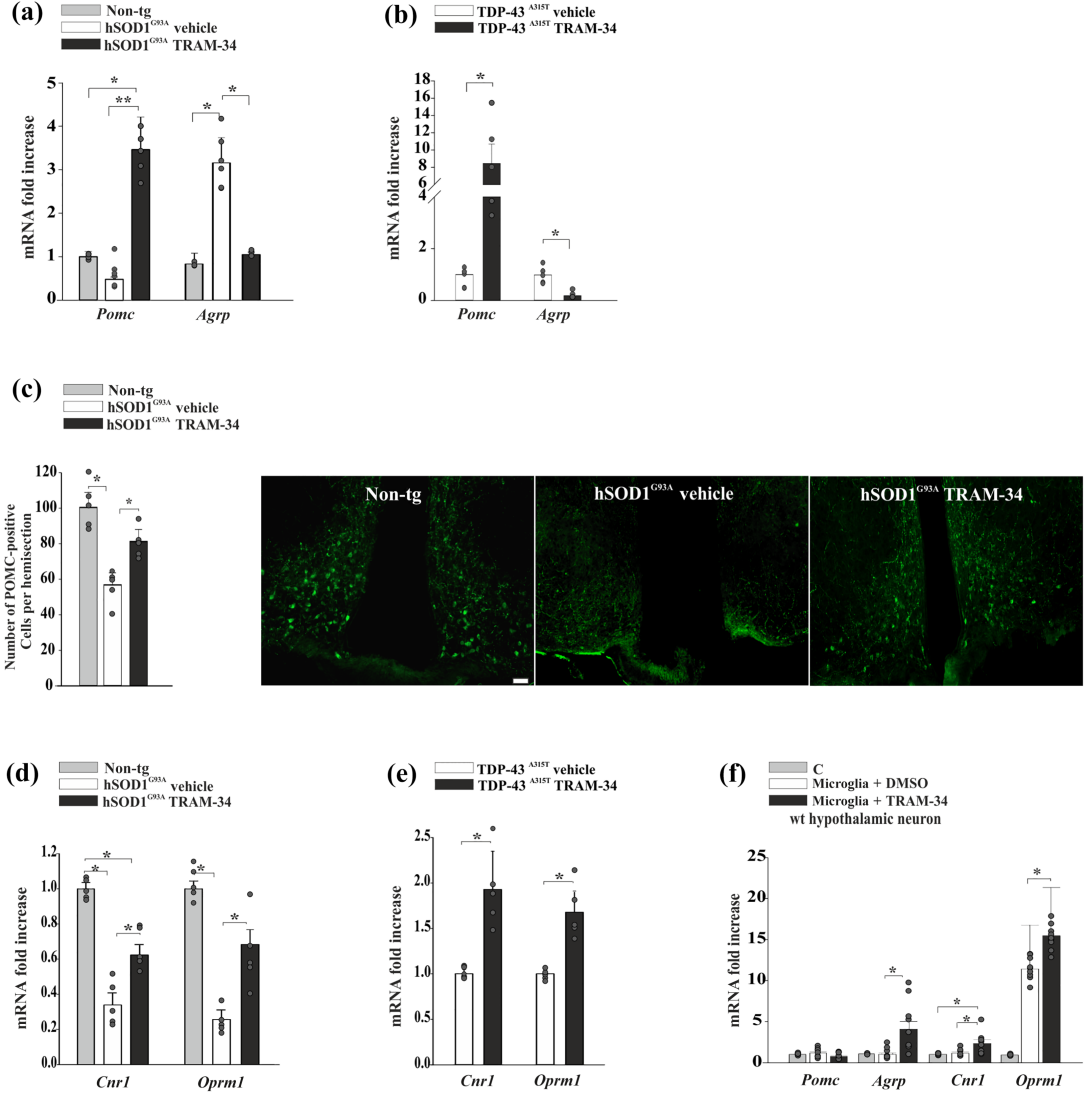
K_Ca_3.1 inhibition changes the expression of genes related to melanocortin signalling in hSOD1^G93A^ and TDP‐43^A315T^ mice. (a) RT‐PCR of *Pomc* and *Agrp* expression in the hypothalamus of non‐tg and hSOD1^G93A^ mice (16 weeks) treated with vehicle or TRAM‐34 (non‐tg, *n* = 5; hSOD1^G93A^ vehicle, *n* = 6; and hSOD1^G93A^ TRAM‐34, *n* = 5 mice). Data are the mean ± SEM. ^*^ *P* < 0.05 one‐way ANOVA. (b) RT‐PCR of *Pomc* and *Agrp* expression in the hypothalamus of TDP‐43^A315T^ mice (9 weeks) treated with vehicle or TRAM‐34 (vehicle, *n* = 5; TRAM‐34, *n* = 5 mice). Data are the mean ± SEM. ^*^ *P* < 0.05 versus vehicle Student's *t*‐test. (c) Quantification of POMC neurons in the hypothalamic arcuate nucleus (ARC) area of non‐tg and hSOD1^G93A^ mice (16 weeks) treated with vehicle or TRAM‐34 (*n* = 5 mice). Data are the mean ± SEM. ^*^ *P* < 0.05, one‐way ANOVA. Representative immunofluorescence images are shown on the right (scale bar = 20 μm). (d) RT‐PCR analysis of hypothalamic *Cnr1* and *Oprm1*, in non‐tg and hSOD1^G93A^ mice (16 weeks) treated with vehicle or TRAM‐34 (*n* = 5). Data are the mean ± SEM. ^*^ *P* < 0.05, one‐way ANOVA. (e) RT‐PCR analysis of hypothalamic *Cnr1* and *Oprm1*, in TDP‐43^A315T^ mice (9 weeks) treated with vehicle or TRAM‐34 (*n* = 5). Data are the mean ± SEM. ^*^ *P* < 0.05 versus vehicle Student's *t*‐test. (f) RT‐PCR analysis of hypothalamic neuropeptides in wt hypothalamic neurons (neurons alone, C) or co‐cultured for 24 h with primary microglia in the presence of vehicle (DMSO) or TRAM‐34 (2.5 μM) (*n* = 3 in triplicate). Data are the mean ± SEM. ^*^ *P* < 0.05, one‐way ANOVA

The hypothalamic control of energy homeostasis is regulated by an intricate network of neuropeptide‐releasing neurons (Yeo & Heisler, 2012). To investigate specific effects induced by K_Ca_3.1 channel blockade on selected hypothalamic neuronal populations, hSOD1^G93A^ and TDP‐43^A315T^ mice were treated with TRAM‐34 or vehicle from the pre‐symptomatic until the disease onset phase. RT‐PCR analysis of the hypothalamic region of these animals shows increased levels of *Pomc* expression in TRAM‐34‐treated hSOD1^G93A^ and TDP‐43^A315T^ mice (Figure 3a,b) (non‐tg: 1.0 ± 0.11, *n* = 5; hSOD1^G93A^ vehicle: 0.75 ± 0.10, *n* = 6; hSOD1^G93A^ TRAM‐34: 3.46 ± 0.75; *n* = 5; TDP‐43^A315T^ vehicle: 1.0 ± 0.13; and TDP‐43^A315T^ TRAM‐34: 8.4 ± 2.25). However, TRAM‐34 treatment reduced *Agrp* (agouti‐related protein gene) expression in hSOD1^G93A^and TDP‐43^A315T^ mice (Figure 3a,b) (non‐tg: 1.0 ± 0.30, *n* = 5; hSOD1^G93A^ vehicle: 3.78 ± 0.69, *n* = 6; hSOD1^G93A^ TRAM‐34: 1.26 ± 0.07; *n* = 5; TDP‐43^A315T^ vehicle: 1.0 ± 0.75; and TDP‐43^A315T^ TRAM‐34: 0.18 ± 0.28). In line with the increased *Pomc* expression in hSOD^G93A^ mice, K_Ca_3.1 inhibition also increased the number of POMC^+^ neurons in the arcuate nucleus of hSOD1^G93A^ mice (Figure 3b) (TRAM‐34: 81.25 ± 6.75%; vehicle: 52.80 ± 5.96% vs. non‐tg 100 ± 8.14%). Interestingly, the expression of some receptors involved in energy homeostasis, such as the hypothalamic CB_1_ receptor (gene *Cnr1*) and μ receptor (gene *Oprm1*) were increased upon K_Ca_3.1 blockade in both ALS mouse models (Figure 3d,e). Trying to identify a direct microglia–hypothalamic neuron communication as a determinant of neuropeptide expression, hypothalamic neuron–microglia co‐culture experiments were performed in the presence of TRAM‐34 (2.5 μM) or vehicle (DMSO). Under these conditions, K_Ca_3.1 inhibition increased the expression level of the orexigenic peptide, *Agrp*, as well as of *Cnr1* and *Oprm1* (Figure 3d). These data indicate that K_Ca_3.1 channels are involved in the regulation of hypothalamic orexigenic and anorexigenic signals in hSOD1^G93A^ mice and suggest that K_Ca_3.1 should be investigated further as a possible target to correct body weight defects in ALS.

### 3.4 K_Ca_3.1 inhibition modulates the release of POMC‐related peptides

**FIGURE 4:**
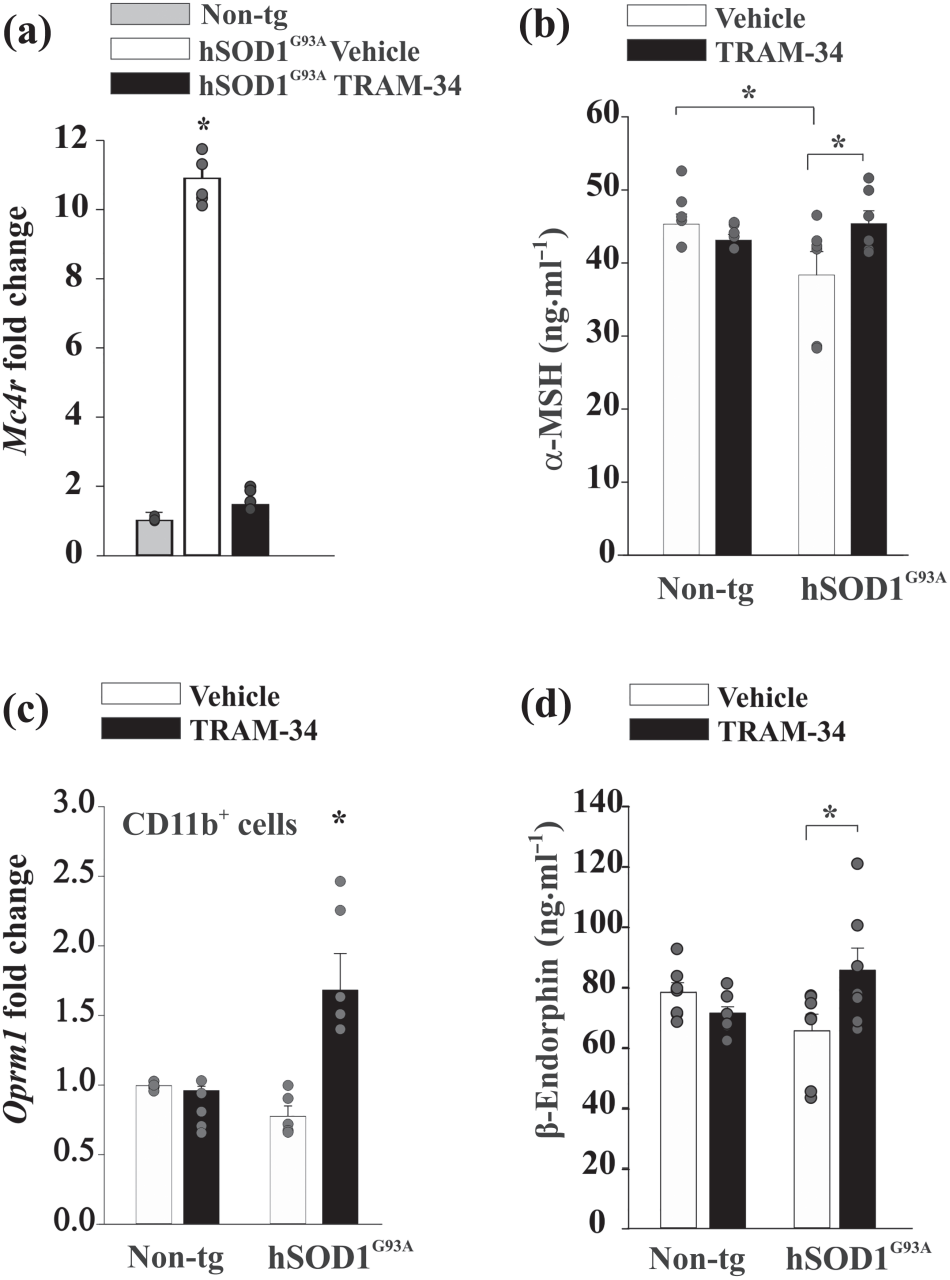
Melanocortin and β‐endorphin pathways are modulated by K_Ca_3.1 blockade in hSOD1^G93A^ mice. (a) RT‐PCR analysis of melanocoritin 4 (MC_4_) receptor gene, *Mc4r,* in the hypothalamus of non‐tg and 16‐week‐old hSOD1^G93A^ mice treated with vehicle or TRAM‐34 (*n* = 5). Data are the mean ± SEM. ^*^ *P* < 0.05, one‐way ANOVA. (b) Analysis of α‐MSH concentrations in CSF samples collected from non‐tg or 16‐week‐old hSOD1^G93A^ mice treated with vehicle or TRAM‐34 (*n* = 8; hSOD1^G93A^ TRAM‐34, *n* = 6). Data are the mean ± SEM. ^*^ *P* < 0.05, two‐way ANOVA. (c) RT‐PCR analysis of μ receptor gene (*Oprm1*) in CD11b^+^ myeloid cells isolated from the hypothalamus of both non‐tg and hSOD1^G93A^ mice (16 weeks) treated with vehicle or TRAM‐34 (*n* = 5). Data are the mean ± SEM. ^*^ *P* < 0.05, one‐way ANOVA. (d) β‐Endorphin levels in CSF samples collected from non‐tg or 16‐week‐old hSOD1^G93A^ mice treated with vehicle or TRAM‐34 (*n* = 7; hSOD1^G93A^ TRAM‐34, *n* = 7). Data are the mean ± SEM. ^*^ *P* < 0.05, two‐way ANOVA

In order to specifically investigate the melanocortin pathway in hSOD1^G93A^ mice, we focused our attention on the neuronal circuits involved in appetite control. To evaluate possible defects in the melanocortin system in hSOD1^G93A^ mice, we first analysed the mRNA levels of *Mc4r* (melanocortin MC_4_ receptor gene), by RT‐PCR analysis, in the hypothalamus of 16‐week‐old hSOD1^G93A^ mice. *Mc4r* mRNA levels were 10‐fold higher in hSOD1^G93A^ mice compared with age‐matched non‐tg mice and were strongly reduced by TRAM‐34 treatment (Figure 4a). We also measured the levels of α‐MSH in the CSF of non‐tg and hSOD1^G93A^ mice upon vehicle and TRAM‐34 treatment. Figure 4b shows that a lower level of α‐MSH expression is observed in hSOD^G93A^ mice and that TRAM‐34 treatment significantly increased this level. Processing of POMC precursor produces several bioactive products in addition to α‐MSH; among them, β‐endorphin is an agonist for μ‐opioid receptors. It has been shown that targeting the cannabinoid system activating CB_1_ receptor, in turn, triggers hypothalamic β‐endorphin release, with effects on body weight (Koch et al., 2015). First, we found that blockade of K_Ca_3.1 channels induced an increase of expression of the μ receptor (*Oprm1*) in CD11b^+^ myeloid cells isolated from the hypothalamus of both non‐tg and hSOD1^G93A^ mice (Figure 4c). Furthermore, we measured β‐endorphin levels in the CSF and the data reported in Figure 4d show that K_Ca_3.1 inhibition significantly increased β‐endorphin levels in the CSF of hSOD1^G93A^ mice (non‐tg, 78.3 ± 3.18 ng·ml^−1^; non‐tg TRAM‐34, 65.5 ± 5.50 ng·ml^−1^; hSOD1^G93A^ vehicle, 71.4 ± 2.13 ng·ml^−1^; and hSOD1^G93A^ TRAM‐34, 85.6 ± 7.35 ng·ml^−1^). Taken together, these results suggest a possible link between microglial K_Ca_3.1 inhibition and β‐endorphin release due to activation of hypothalamic POMC neurons.

### 3.5 K_Ca_3.1 channels trigger cannabinoid–opioid interactions in feeding behaviour

**FIGURE 5:**
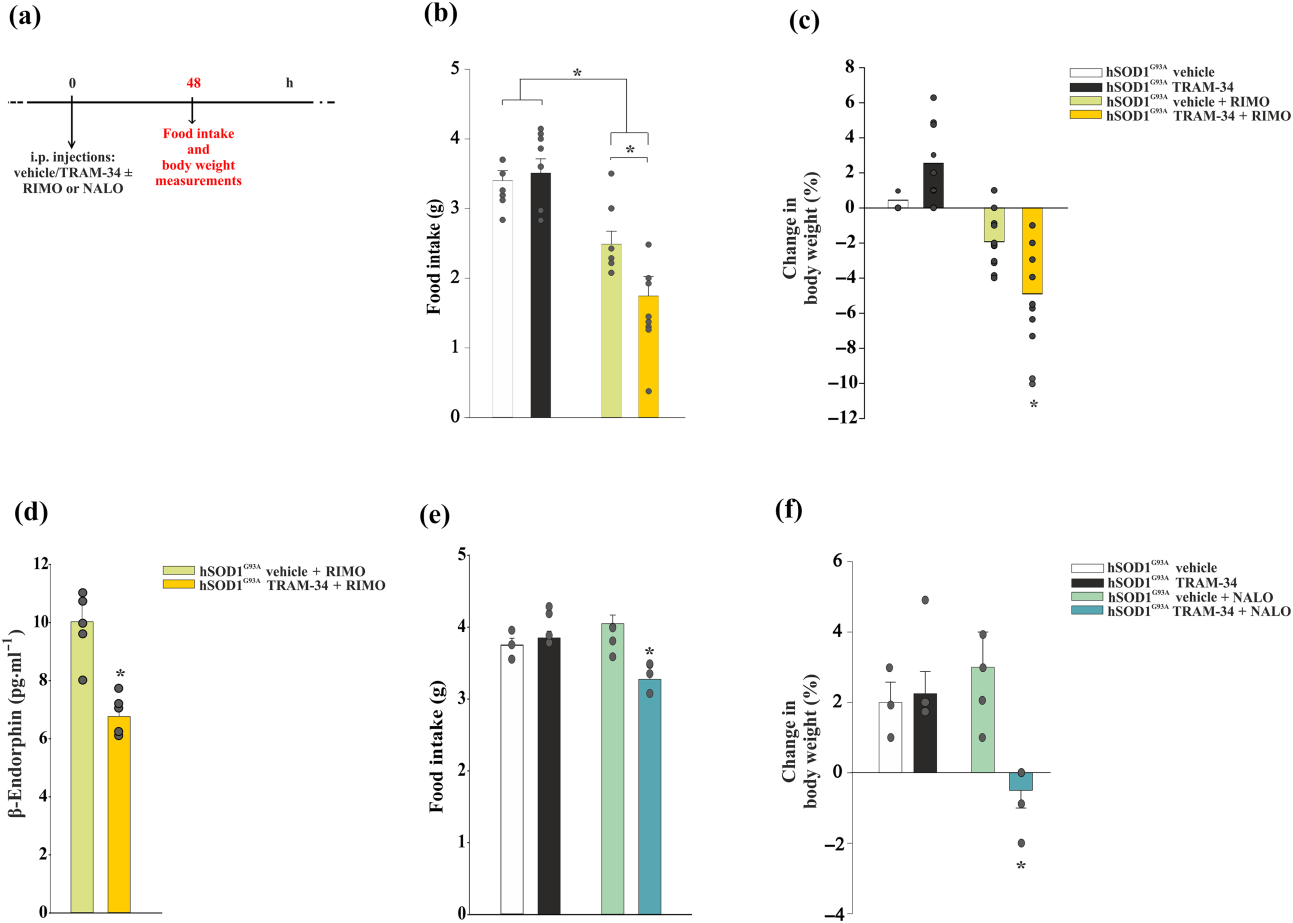
K_Ca_3.1 inhibition drives feeding through cannabinoid signalling. (a) Time scheme adopted for the body weight and food intake experiments. Rimonabant (RIMO) effects on food intake (b) and body weight (c) in hSOD1^G93A^ mice treated with vehicle or TRAM‐34 + RIMO (3 mg·kg^−1^, i.p.) after 48 h (vehicle/TRAM‐34, *n* = 9; vehicle/TRAM‐34 + RIMO, *n* = 11). Data are the mean ± SEM. ^*^ *P* < 0.05 versus controls and hSOD1^G93A^ TRAM‐34 + vehicle, one‐way ANOVA. (d) Quantitative measurement of β‐endorphin content by elisa in the hypothalamus of hSOD1^G93A^mice treated with vehicle or TRAM‐34 + RIMO at 48 h of treatment (vehicle/TRAM‐34, *n* = 5; vehicle/TRAM‐34 + rimonabant, *n* = 5). Data are the mean ± SEM. ^*^ *P* < 0.05 versus vehicle + RIMO, Student's *t*‐test. naloxone (NALO) effects on food intake (e) and body weight (f) in hSOD1^G93A^mice treated with vehicle or TRAM‐34 + NALO (7.5 mg·kg^−1^, i.p.) after 48 h (*n* = 5). Data are the mean ± SEM, versus controls and hSOD1^G93A^ vehicle + NALO. ^*^ *P* < 0.05, one‐way ANOVA

To determine whether the increased expression of CB_1_ receptors triggered by K_Ca_3.1 inhibition is functionally relevant for feeding, mice were treated with the CB_1_ receptor antagonist rimonabant (Kirkham et al., 2002), as shown in Figure 5a. Briefly, mice were treated daily (between 5:00 PM and 6:00 PM) with 120 mg·kg^−1^ of TRAM‐34 or with the same amount of vehicle + rimonabant (3 mg·kg^−1^, i.p.) from 7 weeks of age, for 7 days (Figure S1). This treatment reduced food intake (Figure 5b) and body weight (Figure 5c) with a major effect on TRAM‐34‐treated hSOD1^G93A^ mice after 48 h. We also observed that rimonabant increased the mRNA expression level of *Cnr1* after 2 days (1.60‐fold) and 7 days (1.56‐fold) of treatment (Figure S1d), suggesting that the major effect observed with rimonabant could be due to increased receptor levels. Interestingly, rimonabant induces tolerance to food intake after 4–5 days in both rats and mice, whereas steady reductions in body weight were still observed (Colombo et al., 1998). In our experiments, we also found the development of tolerance after a few days (Figure S1b,c). Rimonabant administration induced a decrease of β‐endorphin secretion in TRAM‐34‐treated hSOD1^G93A^ mice, further confirming the role of this channel in modulating signalling pathways involved in feeding behaviour (Figure 5c). In addition, the μ‐opioid receptor antagonist naloxone (7.5 mg·kg^−1^, i.p.) (Figure S1d) selectively diminished food intake and body weight in TRAM‐34‐treated hSOD1^G93A^ mice already after 48 h (Figure 5d,e). Taken together, these results indicate that K_Ca_3.1 inhibition promotes CB_1_ receptor‐induced feeding, which affects β‐endorphin release by POMC neurons and μ receptor activation.

## 4 DISCUSSION

We show that blockade of K_Ca_3.1 activity induces positive effects on feeding behaviour of hSOD1^G93A^ mice, a model of human familial ALS. We have previously demonstrated that inhibiting K_Ca_3.1 channels with TRAM‐34 reduced the pro‐inflammatory phenotype of spinal cord microglia in hSOD1^G93A^ mice, delaying the appearance of motor signs and lengthening survival time (Cocozza et al., 2018). Recently, hypothalamic inflammation, especially in the glia component, has been associated with a dysregulation of feeding behaviour, raising questions about the role of glia in the modulation of feeding circuits (Benzler et al., 2015). Previous experiments have demonstrated a link between hypothalamic inflammation and the positive energy balance associated with obesity (Valdearcos et al., 2017), whereas others have reported an association of hypothalamic inflammation with the negative energy balance and anorexia–cachexia syndrome (Braun & Marks, 2010). Furthermore, recent findings led to the hypothesis that hypothalamic inflammation impairs neuronal mechanisms of appetite control, whether it is loss of appetite or food overconsumption (Dalvi et al., 2017; Le Thuc et al., 2016). Specifically, neuroinflammation is often associated with ALS, but not yet from metabolic perspective. Ngo et al. (2019) confirmed that appetite loss is prevalent in ALS patients and might also involve factors that extend beyond the physical disability.

Here, we tested the hypothesis that the activity of K_Ca_3.1 channels participates in the hypothalamic neuroinflammation processes that dysregulate feeding behaviours and promote body weight loss, both negatively correlated with survival in ALS (Desport et al., 1999). In accordance with this hypothesis, we observed that in acutely isolated hypothalamic microglia from hSOD1^G93A^ mice, K_Ca_3.1 inhibition increases anti‐inflammatory (*Bdnf*, *Ym1*, *P2yr12* and *Socs3*) and decreases pro‐inflammatory genes (*Inos*, *Tnf‐Α*, *Il‐1β* and *Kcnn4*). Recent transcriptomic studies of microglia in mouse models of Alzheimer's disease, ALS and multiple sclerosis identified subpopulations defined as disease‐associated microglia (DAM) (Keren‐Shaul et al., 2017). Krasemann et al. (2017) identified the TREM2–APOE pathway as a major regulator of microglia phenotypic changes in neurodegenerative diseases and described its induction upon phagocytosis of apoptotic neurons. We here describe that K_Ca_3.1 inhibition increases the expression of genes involved in microglial patrolling activity (*P2ry12*, gene for the P2Y_12_ receptor) and decreases the neurodegeneration‐associated genes, suggesting that K_Ca_3.1 activity, in hSOD^G93A^ mice, contributes to maintain a neurotoxic microglia phenotype. Selectively targeting K_Ca_3.1 may contribute to modulate the microglial phenotype, restoring homeostatic microglia and delaying neurodegeneration. Similarly, morphological analysis of hypothalamic microglia revealed the reacquisition of a more branched phenotype and a wider scanning domain, indicative of a homeostatic function. The reduced inflammatory micro‐environment is accompanied by increased food consumption and weight gain in hSOD1^G93A^ and TDP43^A315T^ mice. Although TRAM‐34‐treated hSOD1^G93A^ mice achieved a similar weight as age‐matched non‐tg mice, food intake remains low irrespective of the amount of weight gained. Dupuis et al. (2004) demonstrated a metabolic deficit in transgenic ALS mice starting from the pre‐symptomatic phase of the disease. These alterations were characterized by reduced adipose tissue accumulation, increased energy expenditure and skeletal muscle hypermetabolism. Administration of highly energetic diet compensated for this energetic imbalance and extended mean survival by 20%. To determine how K_Ca_3.1 inhibition modulates energy expenditure, we performed indirect calorimetry measurements in TRAM‐34 treated and control non‐tg and hSOD1^G93A^ mice. We confirmed metabolic alterations related to energy expenditure in hSOD1^G93A^ mice compared with non‐tg mice and we describe that K_Ca_3.1 blockade restores the alterations of energy metabolism, suggesting that these channels affect metabolic parameters in hSODG93A mice. Specifically, we did not observe differences in energy expenditure among the different groups in the pre‐symptomatic stages (data not shown). Additional studies are needed in more advanced disease stages. Nevertheless, we cannot exclude that alterations of K_Ca_3.1 channel activity also affect energy homeostasis at the cellular level (Kovalenko et al., 2016). Further experiments would be necessary to better understand the mechanisms linking K_Ca_3.1 activity to weight loss in hSODG93A mice. It has been reported that, in obese mice induced by hypercaloric diet, the persistent microglia activation in the mediobasal hypothalamus increases TNF‐α production that, in turn, induces mitochondrial stress in POMC neurons, contributing to POMC neuronal dysfunction (Chun‐Xia et al., 2017). We here observed that TRAM‐34 treatment markedly reduced the expression of *Tnf‐α* of hSOD1^G93A^ mice, suggesting a key role of this channel in the modulation of microglial phenotypes. K_Ca_3.1 blockade increases the expression of *Bdnf* both in hSOD1^G93A^ mice and non‐tg mice, in agreement with the induction of an anti‐inflammatory phenotype. Of note, emerging findings suggest that brain derived neurotrophic factor (BDNF) signalling regulates energy homeostasis, by controlling thermogenesis and feeding behaviour and by modulating glucose metabolism in peripheral tissues (Wang et al., 2020; Xu & Xie, 2016). It would be interesting to explore the role of BDNF in metabolic disorders, including insulin resistance and glucose intolerance that have been reported in ALS patients. The identification of an appropriate strategy to counteract weight loss can increase the survival time of ALS patients, as already demonstrated with a hypercaloric diet (Ludolph et al., 2020; Wills et al., 2014). A few years ago, it has been shown that targeting the cannabinoid system through CB_1_ receptor activation can trigger the release of hypothalamic β‐endorphin, with effects on body weight (Koch et al., 2015). Because the major source of β‐endorphin is *Pomc*, which is decreased in ALS mice, targeting the cannabinoid system to stimulate food intake may not be a successful strategy. Our observation that inhibition of K_Ca_3.1 activity increases the expression of the hypothalamic CB_1_ receptors in both ALS mouse models led us to hypothesize its involvement in β‐endorphin release by POMC neurons and thus in the increase of feeding. In support of this hypothesis, we observed that (i) K_Ca_3.1 inhibition increases β‐endorphin levels in the hypothalamus, likely having orexigenic effects through activation of the μ opioid receptor (Koch et al., 2015) and that (ii), TRAM‐34 treatment increases μ receptor expression in the hypothalamus and, specifically, also in CD11b^+^ myeloid cells. Furthermore, the administration of the inverse CB_1_ agonist rimonabant (rimonabant ‐ to TRAM‐34‐treated hSOD1^G93A^ mice acutely halted the positive stimulation of food intake and body weight gain, before inducing tolerance. In addition, the opioid receptor antagonist naloxone specifically blocks K_Ca_3.1 inhibition‐induced feeding, confirming that cannabinoid–opioid interactions are crucial for the hypothalamic regulation of feeding in TRAM‐34 treated hSOD1^G93A^.

## 5 CONCLUSIONS

In conclusion, our data demonstrate that K_Ca_3.1 blockade promotes positive effects on feeding behaviour of hSOD1^G93A^ mice inducing (i) an anti‐inflammatory phenotype in hypothalamic microglia, (ii) weight gain and increased food consumption, (iii) restored melanocortin tone and (iv), expression of neuropeptides involved in energy homeostasis. These data strongly support the need to identify specific targets to correct the energy metabolism defects observed in ALS mouse models and in patients. In particular, it will be important to investigate the communication pathways among cannabinoids, β‐endorphin and food intake, and the interaction between POMC‐expressing and μ receptor‐expressing neurons. We speculate that possible mediators of this communication could be microglia‐derived microvesicles (MVs). It has been shown that endocannabinoids may be released by microglia‐derived microvesicles, which can activate CB_1_ receptors and influence synaptic communication (Gabrielli et al., 2015). It is important to note that a drug structurally related to TRAM‐34, senicapoc, has been previously found to be safe in humans in Phase I, II and III clinical trials (Ataga et al., 2008, 2011) and would be available for repurposing, and has been deposited by Pfizer for exactly this purpose in the National Center for Advancing Translational Sciences (NCATS) library. To conclude, our data suggest a new, unexpected role for K_Ca_3.1 in the regulation of feeding behaviour, highlighting the importance of counteracting inflammation in the hypothalamus as a target in the fight against weight loss and disease progression in ALS.

## ACKNOWLEDGEMENTS

This work was supported by Progetti di Rilevante Interesse Nazionale (PRIN) 2017 and Associazione Italiana per la Ricerca sul Cancro (AIRC) 2019; Ministero della Salute RF2018 to C.L.; and AIRC 22329 2018 and Agenzia di Ricerca per la Sclerosi Laterale Amiotrofica (ARISLA) NKINALS 2019 to S.G. Open Access Funding provided by Universita degli Studi di Roma La Sapienza within the CRUI‐CARE Agreement. [Correction added on 17 May 2022, after first online publication: CRUI funding statement has been added.]

## AUTHOR CONTRIBUTIONS

C.L. and G.C. contributed to the conception, design and writing of the manuscript. G.C., S.G., M.M., M.L. and A.G. conducted the experiments. H.W. provided critical reagent and contributed to the writing of the manuscript. All authors reviewed and approved the final version of the manuscript.

## CONFLICT OF INTEREST

The authors declare no conflicts of interest.

## DECLARATION OF TRANSPARENCY AND SCIENTIFIC RIGOUR

This Declaration acknowledges that this paper adheres to the principles for transparent reporting and scientific rigour of preclinical research as stated in the *BJP* guidelines for Design & Analysis, Immunoblotting and Immunochemistry and Animal Experimentation and as recommended by funding agencies, publishers and other organizations engaged with supporting research.

## Supporting information


**Figure S1.** K_Ca_3.1 inhibition promotes feeding behaviour by opioid and cannabinoid receptor activation. a: Treatment scheme. Cumulative food intake (b) and body‐weight (c) change of hSOD1^G93A^mice treated with vehicle or TRAM‐34 plus rimonabant (RIMO; 3 mg·kg^‐1^; i.p.) (vehicle/TRAM‐34 n = 9; vehicle/TRAM‐34 plus rimonabant n = 11. (b): Data are the mean ± SEM, *P < 0.05, *P < 0.05 *vs* hSOD1^G93A^ vehicle/TRAM‐34, ^#^ *P* < 0.05 vs hSOD1^G93A^ vehicle + RIMO, one‐way ANOVA; (c): Data are the mean ± SEM, *P < 0.05 *vs* hSOD1^G93A^ vehicle + RIMO, one‐way ANOVA). d: RT‐PCR analysis of *Cnr1* gene expression in the hypothalamus of non‐tg and hSOD1^G93A^ mice treated with TRAM‐34 or the same amount of vehicle plus rimonabant (vehicle/TRAM‐34 n = 5; vehicle/TRAM‐34 plus rimonabant n = 6. Data are the mean ± SEM, *P < 0.05 one‐way ANOVA). e: Treatment scheme. Cumulative food intake (b) and body‐weight (c) change of hSOD1^G93A^mice treated with vehicle or TRAM‐34 plus naloxone (NALO) (7.5 rimonabanti.p.) (vehicle/TRAM‐34 n = 5; vehicle/TRAM‐34 plus rimonabant n = 5. (b): Data are the mean ± SEM, *P < 0.05, *vs* hSOD1^G93A^ vehicle + NALO, one‐way ANOVA; (c): Data are the mean ± SEM, *P < 0.05, *vs* hSOD1^G93A^ vehicle + NALO, one‐way ANOVA).

**Figure S2.** Metabolic effects of K_Ca_3.1 inhibition in hSOD1^G93A^ mice.
a: Mean 12‐h values for *V*o2, *V*co2 and respiratory quotient (RER) in non‐tg and 13 week‐old hSOD1^G93A^ mice treated with vehicle or TRAM‐34 for six days (n = 146 registrations/3 mice per condition). Data are the mean ± SEM, *P < 0.05, two‐way ANOVA).

## Data Availability

The datasets used and/or analysed during the current study are available from the corresponding author on reasonable request.
