# Supplementary figures and images for: The feeding behaviour of Amyotrophic Lateral Sclerosis mouse models is modulated by the Ca2+‐activated KCa3.1 channels
Source: Br J Pharmacol. 2021 Oct 5;178(24):4891–906. doi: 10.1111/bph.15665 (PMC9293222; doi:10.1111/bph.15665)

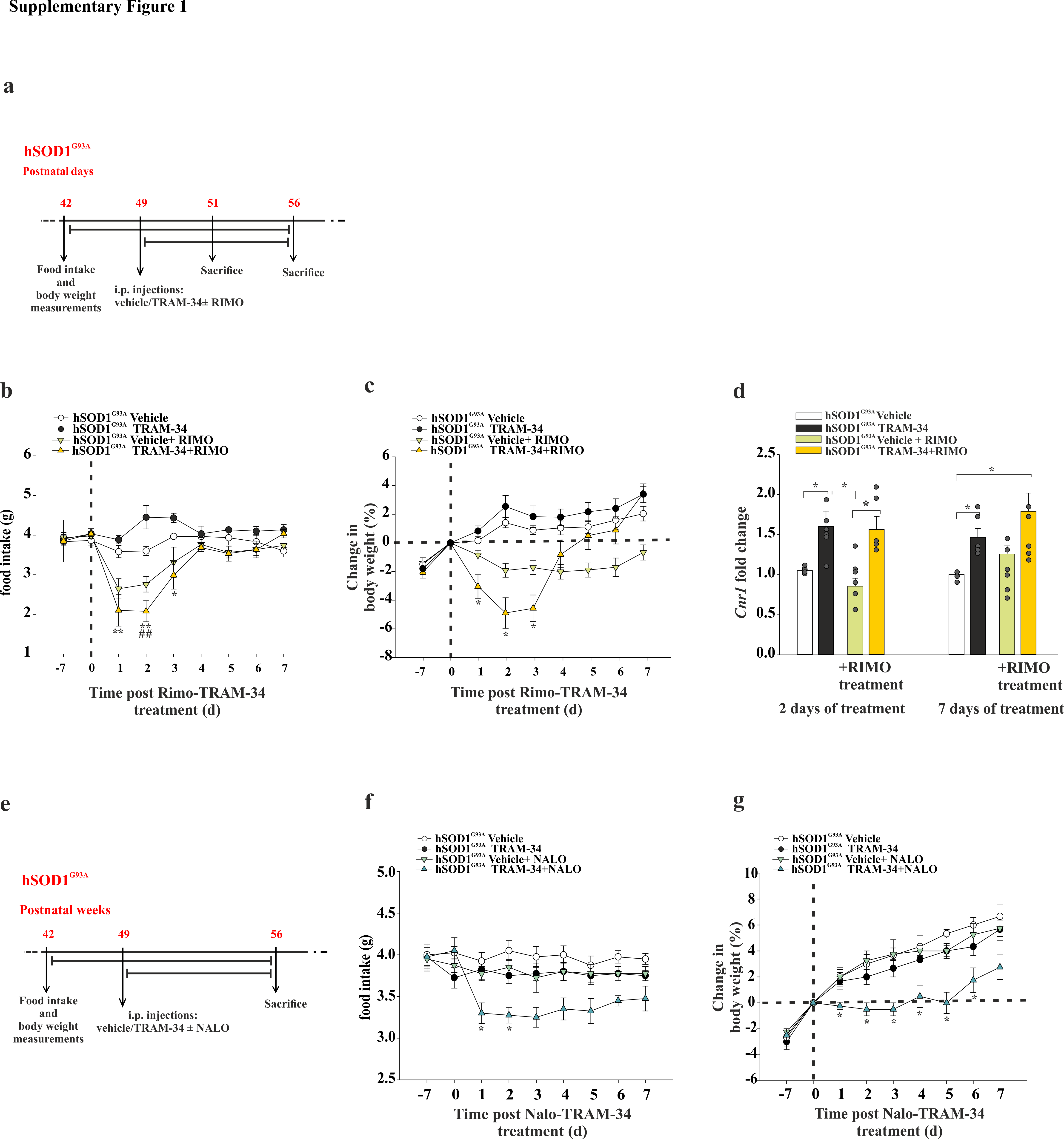

Supplement: Supplementary file 1 — Figure S1. KCa3.1 inhibition promotes feeding behaviour by opioid and cannabinoid receptor activation. a: Treatment scheme. Cumulative food intake (b) and body‐weight (c) change of hSOD1G93Amice treated with vehicle or TRAM‐34 plus rimonabant (RIMO; 3 mg·kg‐1; i.p.) (vehicle/TRAM‐34 n = 9; vehicle/TRAM‐34 plus rimonabant n = 11. (b): Data are the mean ± SEM, *P < 0.05, *P < 0.05 vs hSOD1G93A vehicle/TRAM‐34, # P < 0.05 vs hSOD1G93A vehicle + RIMO, one‐way ANOVA; (c): Data are the mean ± SEM, *P < 0.05 vs hSOD1G93A vehicle + RIMO, one‐way ANOVA). d: RT‐PCR analysis of Cnr1 gene expression in the hypothalamus of non‐tg and hSOD1G93A mice treated with TRAM‐34 or the same amount of vehicle plus rimonabant (vehicle/TRAM‐34 n = 5; vehicle/TRAM‐34 plus rimonabant n = 6. Data are the mean ± SEM, *P < 0.05 one‐way ANOVA). e: Treatment scheme. Cumulative food intake (b) and body‐weight (c) change of hSOD1G93Amice treated with vehicle or TRAM‐34 plus naloxone (NALO) (7.5 rimonabanti.p.) (vehicle/TRAM‐34 n = 5; vehicle/TRAM‐34 plus rimonabant n = 5. (b): Data are the mean ± SEM, *P < 0.05, vs hSOD1G93A vehicle + NALO, one‐way ANOVA; (c): Data are the mean ± SEM, *P < 0.05, vs hSOD1G93A vehicle + NALO, one‐way ANOVA). [file BPH-178-4891-s002.tif]

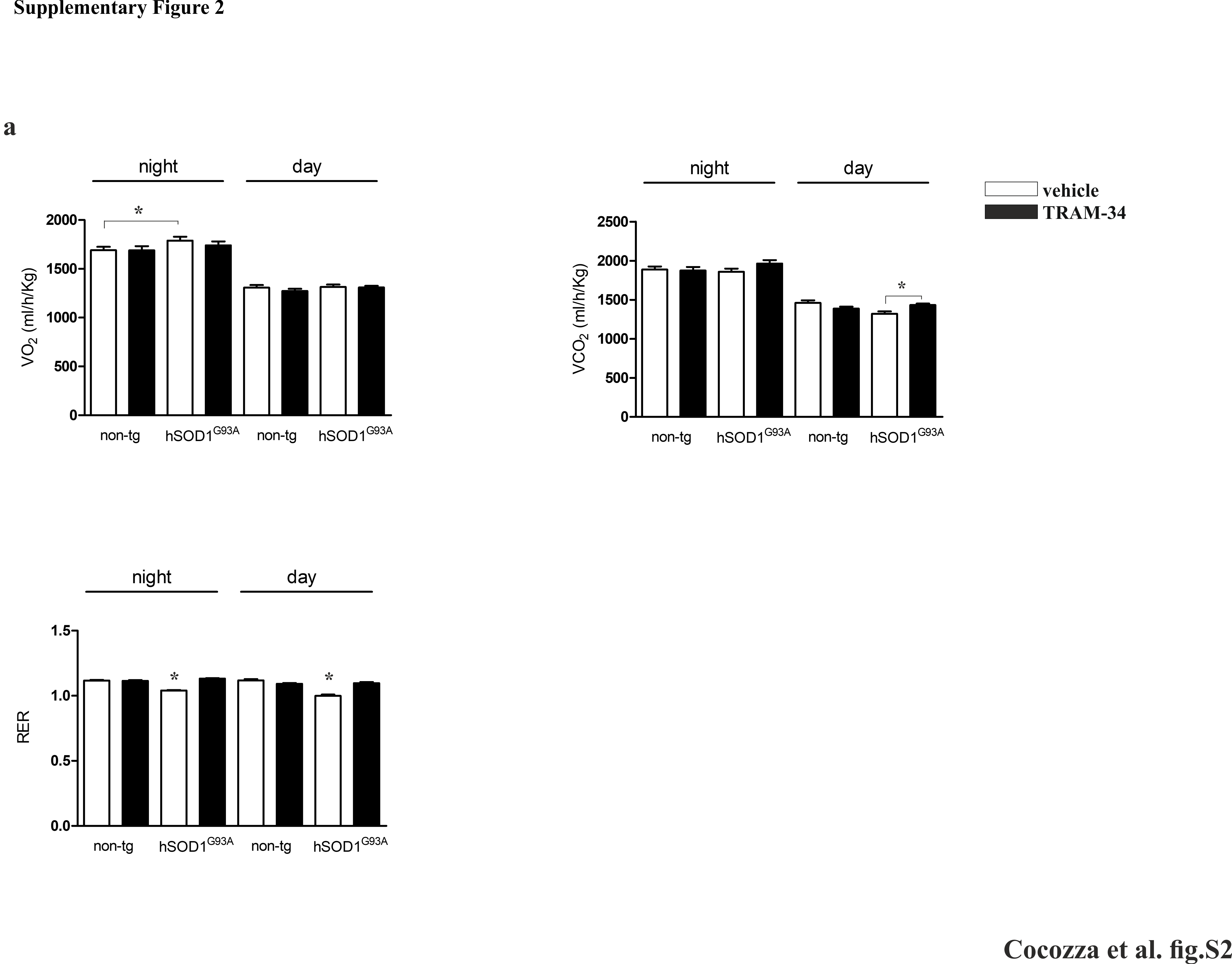

Supplement: Supplementary file 2 — Figure S2. Metabolic effects of KCa3.1 inhibition in hSOD1G93A mice. a: Mean 12‐h values for Vo2, Vco2 and respiratory quotient (RER) in non‐tg and 13 week‐old hSOD1G93A mice treated with vehicle or TRAM‐34 for six days (n = 146 registrations/3 mice per condition). Data are the mean ± SEM, *P < 0.05, two‐way ANOVA). [file BPH-178-4891-s001.tif]
